# Comparison of The Melatonin Preconditioning Efficacy between Bone Marrow and Adipose-Derived Mesenchymal Stem Cells

**DOI:** 10.22074/cellj.2019.5507

**Published:** 2018-08-07

**Authors:** Ali Rafat, Amaneh Mohammadi Roushandeh, Akram Alizadeh, Nasrin Hashemi-Firouzi, Zoleikha Golipoor

**Affiliations:** 1 Department of Anatomical Sciences, School of Medicine, Hamadan University of Medical Sciences, Hamadan, Iran; 2 Medical Biotechnology Research Center, Paramedicine Faculty, Guilan University of Medical Sciences, Rasht, Iran; 3 Department of Tissue Engineering, School of Advanced Technologies, Shahrekord University of Medichal Sciences, Shahrekord, Iran; 4 Neurophysiology Research Center, Hamadan University of Medical Sciences, Hamadan, Iran; 5 Cellular and Molecular Research Center, Faculty of Medicine, Guilan University of Medical Sciences, Rasht, Iran

**Keywords:** Apoptosis, Bone Marrow Mesenchymal Stem Cells, Melatonin, Osteogenesis

## Abstract

**Objective:**

Mesenchymal stem cells (MSC) from various sources have the potentials to positively affect regenerative medicine. Furthermore, pre-conditioning strategies with different agents could improve the efficacy of cell therapy. This study compares the effects of an anti-inflammatory and antioxidant agent, melatonin, on protection of bone marrow-derived MSCs (BMSCs) and adipose tissue-derived MSCs (ADSCs).

**Materials and Methods:**

In this experimental study, rat BMSCs and ADSCs were isolated and expanded. Pre-conditioning was performed with 5 µM melatonin for 24 hours. Cell proliferation and viability were detected by MTT assay. Expression of *BAX*, *BCL2*, melatonin receptors and osteocalcin genes were evaluated by reverse transcriptase-polymerase chain reaction (RT-PCR). Also, apoptosis was detected with tunnel assay. Osteogenic differentiation was analyzed using alizarin red staining.

**Results:**

No significant increase was found in cell viability between BMSCs and ADSCs after melatonin preconditioning. Following melatonin preconditioning, *BAX* expression was significantly down-regulated in both ADSCs and BMSCs (P<0.05), with the difference being more significant in ADSCs compared to BMSCs. *BCL2* expression was increased significantly in both cell types after preconditioning. Metalothionine 1 and Metalothionine 2 were both upregulated significantly in the two cell types (P<0.05). Melatonin increased osteogenesis capability through increasing osteocalcin expression. However, expression of osteocalcin in BMSCs before and after preconditioning was higher than that in ADSCs. On the other hand, melatonin expression in ADSCs was in higher levels than in BMSCs. Melatonin also improved alizarin red concentration significantly in both BMSCs and ADSCs (P<0.05). Alizarin red staining severity increased significantly in ADSCs after preconditioning compared to BMSCs (P<0.05).

**Conclusion:**

Here we have shown that the effects of preconditioning on melatonin expression in ADSCs are higher than those in BMSCs. These findings could be used in adoption of a proper preconditioning protocol based on the sources of MSCs in specific clinical applications, especially in bone regeneration.

## Introduction

In the field of cell therapy and regenerative medicine, bone marrow and adipose tissue are considered as two main sources of mesenchymal stem cells (MSCs) (1-5).

Bone marrow MSCs (BMSCs) and adipose-derived stem cells (ADSCs) present similar properties morphologically and in terms of cell surface antigens (4, 6). On the other hand, they show some significant biological differences like proliferation rate, differentiation capacity, cytokine secretome and chemokine receptor expression (7). ADSCs represent biological advantages in proliferation potentials and immunomodulatory effects, while BMSCs have advantages in osteogenic and chondrogenic differentiation capabilities. Also, in terms of differences in secreted proteins, ADSCs produce basic fibroblast growth factor, interferon-γ, and insulin-like growth factor-1, while BMSCs produce stem cell- derived factor-1 and hepatocyte growth factor (8).

Finding a safe harvesting protocol with low pain for MSC isolation is a challenge in cell therapy. Unlike ADSC isolation, BMSC harvest procedure is invasive and painful for the patients. In addition to the problems associated with cell harvest, the number of isolated cells is low from both sources and *in vitro* expansion of the cells is needed prior to transplantation. Therefore, they are frequently subjected to oxidative stress and other toxic factors within their microenvironment that lead to apoptosis during the harvest, expansion and transplantation processes (9). It is demonstrated that preconditioning with some agents not only can reduces oxidative stress and apoptosis, but also can increase some desired potentials of MSCs (10, 11). Melatonin, a human pineal gland hormone, has anti inflammatory and anti-apoptotic properties (12). It is also a powerful free radical scavenger and activator of cellular antioxidants in various cell types. In addition, melatonin is a safe drug that has been approved by FDA with few side effects and its therapeutic effects have been proven in several human clinical trials (13).

Evidence suggests that melatonin protects human ADSCs from oxidative stress and cell death (9). Previous studies have shown that pretreatment with melatonin can enhance the homing of BMSCs after transplantation (14) and improves therapeutic outcomes of BMSCs in the case of transplantation in liver fibrosis (15). Also, it is suggested that melatonin may contribute significantly in regulation of osteogenic differentiation of MSCs (11). 

Although there are strong evidences to show the cytoprotective effects of melatonin, it is necessary to know its behavior after using as a preconditioning agent. Therefore, the present study is designed to compare preconditioning efficacy of melatonin in BMSCs and ADSCs. 

## Materials and Methods

### Study design 

The present study was designed as an experimental study. The cells were divided into 4 treatment groups. BMSCs with or without melatonin treatment, ADSCs with or without melatonin treatment. Reverse transcriptasepolymerase chain reaction (RT-PCR) was performed for the 4 treatment groups.

### Isolation and expansion of bone marrow mesenchymal stem cells

All animal studies were approved by the Ethical Committee of Hamadan University of Medical Sciences. About 6-8 weeks-old male Wistar rats were euthanized by diethyl ether and their femurs and tibia were removed under sterile conditions. Then, in the long bones proximal and distal ends were cut. Bone marrow was obtained by flushing of a-Minimum Essential Medium (a-MEM, Sigma, USA) containing 1000 U/ml Penicillin through the bones using a syringe (22G needle). The collected bone marrow was centrifuged at 1000×g for 5 minutes. and the pellets were collected. Finally, the harvested cells were cultured at a density of 1.0×10^6^ in each T75 tissue culture flask containing a-MEM with 15% fetal bovine serum (Sigma, USA), 100 U/ml penicillin and 100 µg/ml streptomycin. The medium was refreshed every 3 days. Cells were sub-cultured using trypsin/ ethylenediaminetetraacetic acid (EDTA, Sigma, USA) when they reached 90% confluency. 

### Isolation and expansion of adipose tissue-derived mesenchymal stem cells 

After euthanizing the rats, the white adipose tissue of epididym from each rat was removed in antiseptic conditions. The adipose tissue was warmed in 37°C and then washed two times with phosphate-buffered saline (PBS, Invitrogen, USA) containing 1% Penicillin/ Streptomycin (Invitrogen, USA). To digest the adipose tissue the samples were treated with 0.1% collagenase type I (Gibco, USA) and 1% bovine serum albumin (BSA, dissolved in warm PBS) (Invitrogen, USA). For total digestion and homogenization, the sample was submerged in water bath for 30 minutes. Then, it was centrifuged at 1200 rpm at room temperature for 5 minutes. The supernatant was discarded and the pellet was re-suspended in 1% BSA solution and was centrifuged again in red blood cell (RBC) lysis buffer to remove red blood cells (Kiazist, Iran). Finally, the harvested cells were cultured in DMEM/Ham’s F-12 medium containing 10% Iran. Finally, the harvested cells were cultured in DMEM/Ham’s F-12 medium containing 10% fetal bovine serum (FBS, Gibco, USA) and 1% Penicillin/Streptomycin at 37°C and 5% CO_2_. The medium was changed every 3 days and the cells were sub-cultivated using trypsin/EDTA (Sigma, USA) at 90% confluency. 

### Multi-lineage differentiation of BMSCs and ADSCs 

Passage 2 cells were cultured in DMED-Low glucose for 3 days at a density of 5000 cells/cm^2^. Then the medium was replaced with differentiation media. The osteogenic differentiation medium contained aMEM, 10% FBS, 2 mM L-glutamine, 100 U/ml penicillin and 100 mg/ml streptomycin, 10 nM dexamethasone, 50 mg/ml L-ascorbic acid and 10 mM b-glycerophosphate. The adipogenic medium consisted of aMEM, 10% FBS, 2 mM L-glutamine, 100 U/ml penicillin and 100 mg/ ml streptomycin, 10 nM dexamethasone, 200 mg/ml indomethacin, 5 mg/ml insulin and 0.5 mM IBMX. Each of the media were refreshed every 3-4 days. After 21 days, osteogenic and adipogenic differentiations were detected by alizarin red and oil red O staining, respectively. 

### Melatonin preconditioning of BMSCs and ADSCs 

Passage 5 cells were cultured in T-75 flasks. After 24 hours, the cells were pretreated with 5µM melatonin (Sigma, USA) for 24 hours. The melatonin solution was prepared by dissolving it in ethanol at a concentration of 1.15 µg/ml (14). Then the pretreated cells were washed to remove the melatonin solution and were cultured in 96well plates at a density of 104 for further experiments.

### Cell viability assay 

The 3-(4, 5-Dimethylthiazol- 2-yl)-2, 5-diphenyltetrazolium bromide (MTT, Sigma, USA) test represents the mitochondrial metabolic activity in cell culture, which indicates the number of viable cells. Briefly, the cells were cultivated into a 96-well plate at a density of 1.0×10^6^/well. After washing with PBS, 100 µl of culture medium containing 50 µl MTT reagent was added to each well. Following incubation in the incubator at 37°C and 5% CO_2_ for 1 hour, 200 µl dimethyl sulfoxide (DMSO) was added to the wells and the absorption of the media was measured by ELISA Reader at 630 nm. 

### Reverses transcriptase-polymerase chain reaction

Total RNA was extracted from the cells using RNA extraction solution (RNX™, Cinnagen, Iran). The quantity and quality of the extracted RNA were checked using nanodrop (Thermo Fisher Scientific, USA) and electrophoresis, respectively. cDNA was synthesized from 5 µg total RNA using a Fermentas kit (Fermentas, Canada) according to the manufacturer’s manuals. Then, 25 µl of PCR cocktail, containing 0.2 pM of each primer (forward and reverse) (Table 1), 0.3 mM dNTP, 1.5 mM MgCl2, 1U taq DNA polymerase, and 1×PCR buffer (Fermentas, Canada) was used for each sample. The PCR reactions were conducted in a thermocycler (Bio-rad, USA) with the following program: 94°C for 5 minutes, 35 cycles at 94°C for 45 seconds, 55°C for 45 seconds, 72°C for 45 seconds, and a final extension at 74°C for 10 minutes. Ten µg of the PCR product were separated, run on a 1.5% agarose gel, and stained with SYBR safe.

**Table 1:** Primers and expected length of products


Primer	Sequence (5΄-3΄)	Length (bp)

*β-actin*	F: CTCTGTGTGGATTGGTGGCT	219
	R: CGCAGCTCAGTAACAGTCCG	
*Melatonin Receptor1(MT1)*	F: CGGACAGCAAACCCAAACT	152
	R: AACTAGCCACGAAGAGCCAC	
*MT2*	F: TGACCTGTTACTGAATGTTGCC	199
	R: GAACTGCGATTTCTGGGTTAC	
*BAX*	F: AACAACATGGAGCTGCAGAGG	304
	R: GAAGTTGCCGTCTGCAAACAT	
*BCL-2*	F: TGACTTCTCTCGTCGCTACC	116
	R: CACAATCCTCCCCCAGTTCA	
*Osteocalcin*	F: AGGACCCTCTCTCTGCTAC	138
	R: AACGGTGGTGCCATAGATGC	


### Apoptosis detection

The cells were grown in a 96-well plate and pretreated with 5 µM melatonin for 24 hours. Following the treatment, the cells were rinsed with PBS and fixed in 4% paraformaldehyde. The endogenous peroxidase activity was blocked by methanol followed by cell permeabilization with a cocktail of 1 g/L TritonX-100 in 0.1% sodium citrate. TUNEL reaction solution and Converter-POD were added to the cells according to the kit manual. The reaction was developed by 3, 3'-diaminobenzidine (DAB) and cell apoptosis was observed under light microscope (Ziess Germany) with 400 magnification.

### Osteogenesis analysis using alizarin red concentration 

To investigate the effects of melatonin on osteogenic differentiation potentials of the stem cells before and after pretreatment with melatonin, osteogenic differentiation was induced and after 21 days osteogenesis was analyzed using alizarin red.

### Statistical analysis

Our data was analyzed by two-way ANOVA, followed by Bonferroni post hoc test, and was presented as the mean ± SD. P<0.05 was considered as significant. All experiments were performed in at least triplicates.

## Results

### BMSCs were expanded easily and had multi-lineage differentiation potentials

**Fig.1:**
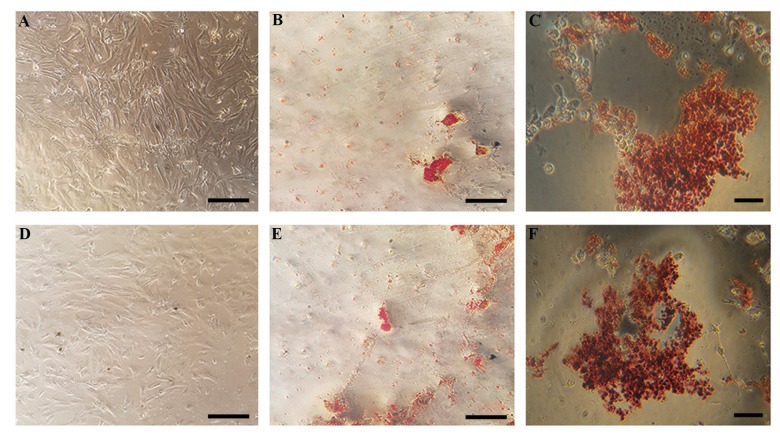
The morphology of undifferentiated and differentiated BMSCs and ADSCs. **A, D.** Undifferentiated BMSCs and ADSCs, display a flattened fibroblast-like morphology under phase-contrast microscopy. Alizarin red staining of the B. BMSCs and E. ADSCs after culturing for 21 days in osteogenic differentiation medium. Oil-red-O staining of the **C.** BMSCs and **F.** ADSCs after culturing for 5 days in adipogenic differentiation medium (scale bar: 200 µm). BMSCs; Bone marrow mesenchymal stem cells and ADSCs; Adipose tissue-derived mesenchymal stem cells.

The isolated BMSCs were adhered to the culture dish after 24-48 hours and their primary round form changed to a more spindle-like shape (Fig .1A). Deposition of calcium and alizarin red staining after 21 days of culture showed that the cells differentiated into osteoblasts in differentiation medium (Fig .1B). Similarly, after 5 days in culture, cells that were plated in adipogenic differentiation medium successfully stained with oil- red-O, demonstrating adipogenic potentials of the harvested cells prior to tratment (Fig .1C). 

### Isolation, expansion and multi-lineage differentiation potential of ADSCs

The isolated cells from rat adipose tissue adhered to tissue culture flasks within 48-72 hours after adhesion they formed spindle-like shapes (Fig .1D). Since the first passage, every 2-3 days the cells grew to become confluent in the flasks and needed to be passaged. The isolated cells deposited calcium and stained red (Fig .1C) after 21 days in osteogenic differentiation medium. The cells in adipogenic differentiation medium also confirmed adipogenesis by staining with oil-red-O staining. These cells were cultured for 5 days in the differentiation medium (Fig .1D).

### Melatonin increased cell viability independently from the cell origin

**Fig.2:**
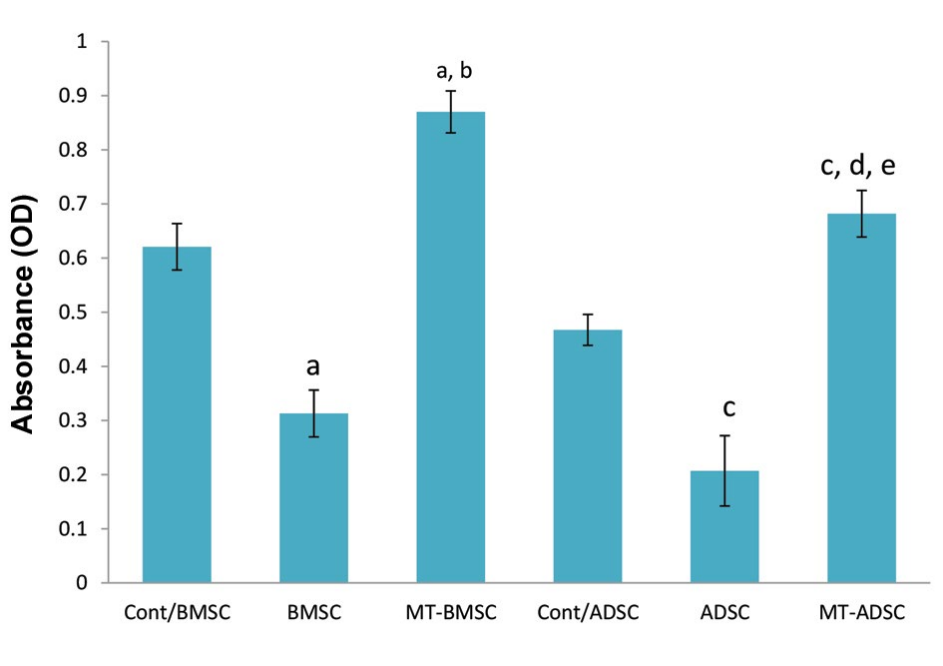
Viability of the cells pretreated with melatonin in BMSCs and ADSCs after culturing them in osteogenic medium. a; P<0.001, compare to Cont/BMSC (control BMSCs: BMSCs were cultured without differentiation medium), b; P<0.05, compare to BMSC, c; P<0.001, compare to Cont/ADSC (control ADSC: ADSC were cultured without differentiation medium), d; P<0.05, compare to ADSC, e; P<0.05, compare to MT-BMSC, BMSCs; Bone marrow mesenchymal stem cells, ADSCs; Adipose tissue-derived mesenchymal stem cells, and MT; Melatonin.

MTT assay analysis showed that pretreatment of the cells with melatonin increased their viability in both BMSCs and ADSCs after being cultured in osteogenesis medium. Although, there were significant differences between the melatonin groups and the controls, no differences were found in cell viability between BMSCs and ADSCs. It seems that melatonin increase cell proliferation independently from the source or origin of the cells (Fig .2). 

### Gene expression profile changes after melatonin preconditioning in BMSCs and ADSCs

**Fig.3:**
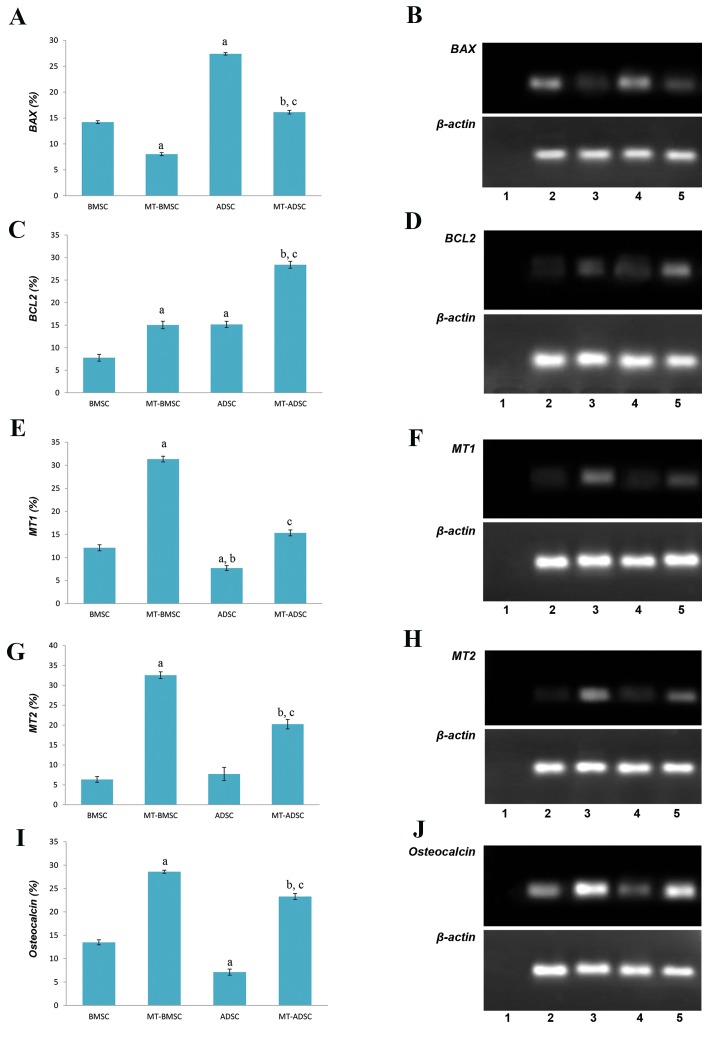
The graph and Electrophotograms of RT-PCR product. **A, B.** *BAX* expression of BMSCs, MT-BMSCs, ADSC, MT-ADSC, **C, D.** *BCL2* expression of BMSCs, MT-BMSCs, ADSC, MT-ADSC (a; Compare to BMSC, b; Compare to ADSC, c; Compare to MT-BMSC), **F, H.** Gene expression of MT1 and MT2, **E, G.** The graph demonstrates MT1 (a; Compare to BMSC, b; Compare to MT-ADSC, c; Compare to MT-BMSC), MT2 (a; Compare to BMSC, b; Compare to ADSC, c; Compare to MT-BMSC) of BMSCs, MT-BMSCs, ADSC, MT-ADSC presents osteocalcinexpression level extracted from BMSCs, MT-BMSCs, ADSC, MT-ADSC (a; Compare to BMSC, b; Compare to ADSC, c; Compare to MT-BMSC). Negative control of RT-PCR: (H_2_O) (Lane 1), BMSC (Lane 2), MT-BMSC (Lane 3), ADSC (Lane 4), MT-ADSC (Lane 5) (P<0.05). RT-PCR; Reverse transcriptase-polymerase chain reaction, BMSCs; Bone marrow mesenchymal stem cells, ADSCs; Adipose tissue-derived mesenchymal stem cells, and MT; Melatonin.

Gene expression profile of MSCs after melatonin pretreatment for *BAX, BCL2, MT1, MT2* and osteocalcin were analyzed using RT-PCR. Our results indicated that melatonin decreased *BAX* expression, as a pro-apoptotic gene, significantly in BMSCs and ADSCs after 24 hours of pretreatment, but it was less significant in ADSCs (Fig .3A, B). Also, melatonin upregulated expression of the anti-apoptotic gene *BCL2* significantly in both cell types. However, the expression was slightly higher in ADSCs (Fig .3C, D). The expression of melatonin receptors (*MT1* and *MT2*) was detected in both of BMSCs and ADSCs. It was found that after melatonin preconditioning, both *MT1* and *MT2* were upregulated significantly in the two cell types. However, their increase was higher in BMSCs than in ADSCs but was not significant (P>0.05, Fig .3E-H). 

After 3 weeks our findings indicated that pretreatment with melatonin increased the expression of osteoblast cell marker, osteocalcin, in both BMSCs and ADSCs. Although the expression of osteocalcin in BMSCs before and after preconditioning with melatonin was higher than that in ADSCs, as a result of melatonin treatment osteocalcin expression increased more significantly in ADSCs compared to BMSCs (Fig .3I, J).

### Melatonin exerts its protective properties through suppression of apoptosis

**Fig.4:**
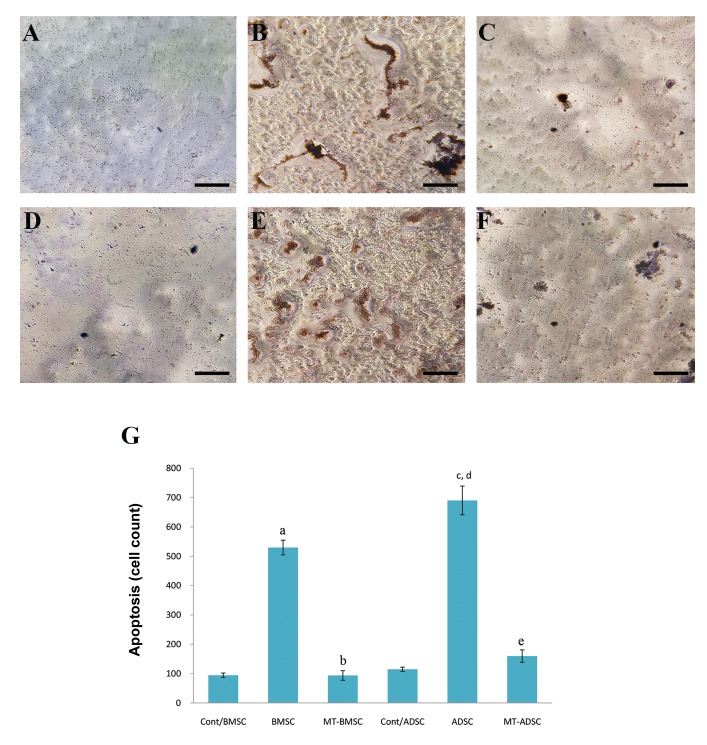
Cell death was detected by TUNEL assay. The apoptotic cells presented their morphology by round shape and brown nuclei. **A.** Control, **B.** Apoptotic cells before pretreatment with melatonin, **C.** Apoptotic BMSCs after pretreatment with melatonin, **D.** Control, **E.** The cells before pretreatment with melatonin, **F.** ADSCs after pretreatment with melatonin, and **G.** The graph shows apoptotic cell numbers before and after pretreatment with melatonin. Melatonin decreased apoptotic cells in ADSCs more than in MSCs (scale bar: 200 µm). a; Compare to Cont/BMSC, b; Compare to BMSC, c; Compare to Cont/ADSC, d; Compare to MT-ADSC, e; Compare to MT-BMSC (P<0.05), BMSCs; Bone marrow mesenchymal stem cells, ADSCs; Adipose tissue-derived mesenchymal stem cells, and MT; Melatonin.

Cell death detection was performed to know whether melatonin might decrease apoptosis in BMSCs and ADSCs. Our findings showed that melatonin reduced apoptosis in the BMSCs and ADSCs significantly after osteogenesis, but its efficiency was more in ADSCs compared to BMSCs (Fig .4). 

### Osteogenic differentiation potentials of melatonin dependent on the source of MSCs

**Fig.5:**
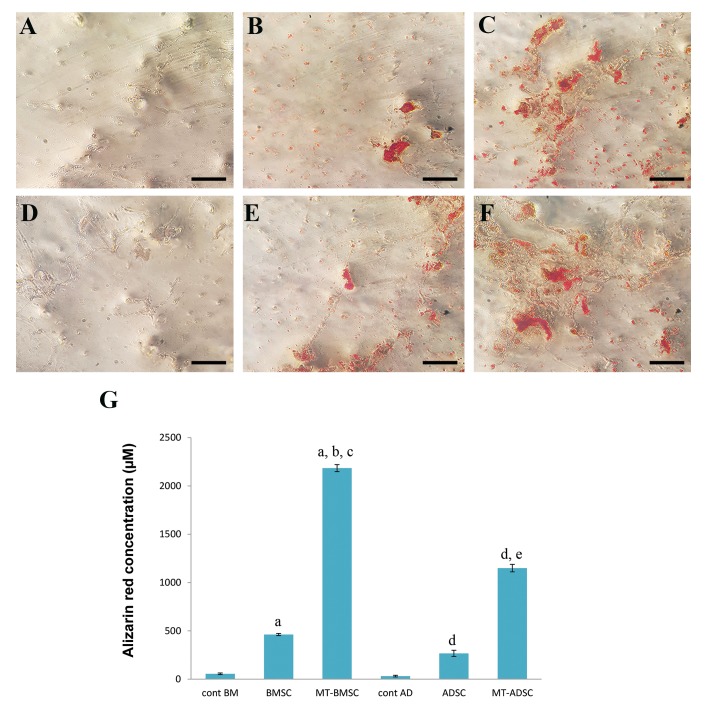
Alizarin red staining for mineral deposition after osteogenic differentiation before and after preconditioning with melatonin. **A.** Control/BMSCs, **B.** BMSCs, **C.** MT-BMSCs, **D.** Control/ADSCs, **E.** ADSCs, **F.** MT-ADSCs after days, **G.** The graph shows Alizarin Red concentration in BMSCs and ADSCs before and after preconditioningwith melatonin. The concentration of alizarin red increased significantly after preconditioning in both cell types, but, more significantly in BMSCs. BMSCs; Bone marrow mesenchymal stem cells, ADSCs; Adipose tissue-derived mesenchymal stem cells, a; Compare to Cont/BMSCs, b; Compare to BMSCs, c; Compare to MT-ADSCs, d; Compare to cont/ADSCs, and e; Compare to ADSCs (P<0.05).

Before and after preconditioning with melatonin, both BMSCs and ADSCs were induced for osteogenic differentiation. After 21 days, the cells were stained with alizarin red and quantitative analysis of alizarin red concentration was performed. As showed in Figure 5, pretreatment with melatonin increased alizarin red concentration significantly in both BMSCs and ADSCs (P<0.05). The increase of alizarin red concentration in ADSCs after preconditioning with melatonin was significantly higher than that in BMSCs (P<0.05). 

## Discussion

The present study was designed to analyze and compare preconditioning efficacy of melatonin in BMSCs and ADSCs as two important sources of stem cells for cell therapy and regenerative medicine.

Our findings are in agreement with previous studies, which demonstrated that melatonin is a potent preconditioning agent for BMSCs and ADSCs (15). Based on our findings, melatonin increases cell viability and inhibits apoptosis, with a higher efficacy in ADSCs compared to BMSCs. Preconditioning with melatonin increases cell viability approximately equally in both cell types, but it suppresses apoptosis in ADSCs more significantly than in BMSCs. Also down-regulation of *BAX* and up-regulation of *BCL2* in ADSCs are significantly more than those in BMSCs. The expression of MT1 and MT2 in BMSCs is significantly higher than that in ADSCs. These findings confirm the previous findings, in which melatonin represented its protective effects via both receptor-mediated and receptor- independent mechanisms (11). 

It is clear that, with the induction of specific melatonin receptors, the antioxidant enzymes, such as catalase and superoxide dismutase-1, are overexpressed, therefore increasing the MSC resistance to hydrogen peroxide- dependent apoptosis (15). 

It has been documented that melatonin has receptor- mediated protective potentials, which result in improved MSC survival (15, 16) and reduced apoptosis (11, 15). It is reported that pretreatment with melatonin has cytoprotective potentials against H_2_O_2_ toxicity and increases MSC viability through an increase in antioxidants capacity, a decline in apoptosis and secretion of inflammatory cytokines (17).

Han and his colleagues have shown that melatonin can increase the therapeutic efficiency of MSCs through activation of antioxidant induction pathways, such as silent information regulator 1 (SIRT1), and upregulation of anti-apoptotic genes (18).

In another study, melatonin protected ADSCs from ROS and improved their therapeutic efficiency in a rat model of myocardial infarction (19). Also, *ex vivo* pretreatment with melatonin enhanced the viability, proangiogenic/ mitogenic activity and efficiency of transplanted MSCs in ischemic kidneys.

Our study demonstrates that melatonin increases osteogenic differentiation potentials of BMSCs and ADSCs, while efficacy of melatonin in osteogenic differentiation of ADSCs is higher than that in BMSCs. In concurrent with our findings, other studies have confirmed the capacity of melatonin in improving bone growth through development in osteoblast cell differentiation and functional competence (20).

It is also demonstrated in other studies that melatonin could regulate the osteogenic differentiation of MSCs through interactions with molecules such as bone- secreted protein (BSP), alkaline phosphatases (ALP) and osteopontin (21). 

Melatonin preconditioning could enhance ADSC and BMSC viability, and on the other hand, decline the number of apoptotic cells. It also improves osteogenic differentiation of these cells. Since there are promising reports about increasing cell therapy outcomes using melatonin (15, 16), further studies should be conducted on comparison of different cell types in response to melatonin administration. In addition, the optimal dose and time of melatonin application with regards to the sources of MSCs should be investigated.

## Conclusion

Our study demonstrated that melatonin preconditioning promotes BMSC and ADSC survival, reduces apoptosis and has positive effects on osteogenic differentiation potentials *in vitro*. Also these results showed that preconditioning affects melatonin expression in ADSCs in a higher level than that in BMSCs. This result can be used in establishing a proper preconditioning protocol for specific MSCs used in clinical applications, especially for bone formation.
